# Cytokines and Neurodegeneration in Epileptogenesis

**DOI:** 10.3390/brainsci12030380

**Published:** 2022-03-12

**Authors:** Pawel Wolinski, Dominika Ksiazek-Winiarek, Andrzej Glabinski

**Affiliations:** Department of Neurology and Stroke, Medical University of Lodz, 90-549 Lodz, Poland; pawel.wolinski@gmail.com (P.W.); dominika.ksiazek@umed.lodz.pl (D.K.-W.)

**Keywords:** epilepsy, IL1β, CXCL2, CXCL12, CCL5, kainic-acid epilepsy model

## Abstract

Epilepsy is a common brain disorder characterized by a heterogenous etiology. Its main features are recurrent seizures. Despite many clinical studies, about 30% of cases are refractory to treatment. Recent studies suggested the important role of immune-system elements in its pathogenesis. It was suggested that a deregulated inflammatory process may lead to aberrant neural connectivity and the hyperexcitability of the neuronal network. The aim of our study was the analysis of the expression of inflammatory mediators in a mouse model of epilepsy and their impact on the neurodegeneration process located in the brain. We used the KA-induced model of epilepsy in SJL/J mice and performed the analysis of gene expression and protein levels. We observed the upregulation of IL1β and CXCL12 in the early phase of KA-induced epilepsy and elevated levels of CCL5 at a later time point, compared with control animals. The most important result obtained in our study is the elevation of CXCL2 expression at both studied time points and its correlation with the neurodegeneration observed in mouse brain. Increasing experimental and clinical data suggest the influence of peripheral inflammation on epileptogenesis. Thus, studies focused on the molecular markers of neuroinflammation are of great value and may help deepen our knowledge about epilepsy, leading to the discovery of new drugs.

## 1. Introduction

Epilepsy is one of the most common chronic brain disorders, affecting about 65 million people worldwide [1]. This disease is characterized by recurrent seizures caused by the abnormal hypersynchrony of neurons. The etiology of epilepsy is heterogenous. Although epilepsy is very common, its pathophysiology is unknown in most cases [2]. Moreover, despite the variety of anti-epileptic medications, about 30% of cases are refractory to treatment and may require the neurosurgical resection of epileptogenic foci to ameliorate seizure recurrence [3]. Epilepsy is often associated with neurological comorbidities, e.g., cognitive decline, anxiety and depression, as well as psychiatric disorders such as autism-spectrum disorders [1].

The increasing body of clinical and experimental evidence suggests that epilepsy is also associated with non-neuronal alterations. Such phenomena may play an important role in the initiation and maintenance of epileptic activity [4]. In recent years, results from various studies suggest that there is a complex relationship between epilepsy and the immune system. Alterations in cytokine expression and immune-cell functions were observed in epileptic patients as well as in animal models [5,6,7]. Moreover, it was suggested that there is a link among inflammatory process, increased permeability of the blood–brain barrier (BBB) and various epileptic syndromes. The interaction between brain endothelium and leukocytes and the subsequent leukocyte recruitment into the brain seem to play important roles in the epileptogenic cascade [8]. Inflammatory mediators activate various signaling pathways via their corresponding receptors located on different brain cells [9]. In pathological conditions, such interaction could lead to neuronal damage, which can manifest clinically, depending on the affected brain region [10]. Although the precise mechanism of epileptogenesis is unknown, it was postulated that a focal or systemic deregulated inflammatory process results in aberrant neural connectivity and in the hyper-excitability of the neuronal network, which stimulate the onset of epilepsy [11,12].

Several inflammatory mediators, such as pro-inflammatory cytokines (IL-1β, IL-6, TNF-α) and transcriptional factor NFκB, were detected in brain tissue surgically resected from patients with temporal-lobe epilepsy (TLE) [13]. Moreover, various animal models of epilepsy were shown to trigger rapid inflammatory responses in brain areas associated with the onset and spread of epileptic activity [14]. Moreover, seizures may induce the expression of cytokines, which, in turn, influence the pathogenesis and course of epilepsy [15,16,17]. A growing body of experimental data suggests that there is an array of cytokines involved in epilepsy pathogenesis; however, their precise role is not clear [17].

Animal models of acquired or genetic epilepsy are valuable for the study of the pathomechanisms of this disorder, including the role of neuroinflammation in seizure generation and recurrence [1]. One of the frequently used animal models of epilepsy is the model induced by administration of kainic acid (KA), which leads to excitotoxic neurodegeneration. KA is a strong functional selective agonist of kainate ionotropic glutamate receptors and a partial agonist of AMPA/KA receptors. The increased activation of these receptors results in the excitotoxic process [18,19]. This phenomenon is observed in various diseases, such as stroke, epilepsy and multiple sclerosis [20,21,22]. The intraperitoneal injection of KA induces damage in neurons located within different brain regions. The neuronal damage results from limbic seizures and elevated glutamate release, which triggers excitotoxicity. Seizures present during the first few hours after KA administration and neuronal damage is detected as early as 4 h after injection [18,23].

The aim of our study is to analyze the expression of selected inflammatory mediators in the brain of mice using a KA-induced epilepsy model and to assess their impact on the neurodegeneration process in the brain.

## 2. Material and Methods

### 2.1. Animals

In all experiments, 8–12-week-old female SJL/J mice (*n* = five for each time point) were used. All animals were housed at the animal facility of Medical University of Lodz, Lodz, Poland, under standard conditions. All animals were used in accordance with the Institutional Animal Care and Use Guidelines. All experiments in this study were approved by the Local Ethics Committee for Affairs Experiments on Animals. Female SJL/J mice were used in this study because the brain immunology of this strain of mice is well known, which we consider very helpful for studying inflammatory mediators in KA-induced animal models of epilepsy. The KA-induced model of epilepsy in SJL mice was previously used by Tanaka et al.; this model showed a neuropathology similar to that of well-established experimental epilepsy in C57BL/6 mice [24]. Moreover, we have a very long experience in using these animals in experimental autoimmune encephalomyelitis (EAE)—an animal model of multiple sclerosis [25].

### 2.2. Induction of Kainic-Acid-Induced Epilepsy Model

The animal epilepsy model was induced by stereotactic, intracerebral injection of KA (KA; Sigma-Aldrich, Poznan, Poland). KA is a substance with a selective neurotoxic activity that is specific to hippocampal neurons. KA was injected once in the amount of 0.5 μg in 1 μL of PBS per mouse. Prior to injection, each mouse was anaesthetized with mixture of ketamine (1.15 mg; Biowet, Pulawy, Poland) and xylazine (0.1 mg; Biowet, Pulawy, Poland). After complete anaesthetization, mice were placed in a stereotactic frame (David Kopf Instruments, Los Angeles, CA, USA), the skin on the head was cut and a small hole in the skull was drilled. KA was administered with a Hamilton syringe (32G needle) (Hamilton Company, Bonaduz, GR, Switzerland). The site of injection (A—2 mm; L—1.2 mm; D—2.5 mm) was selected using the stereotactic atlas “The Mouse Brain in Stereotaxic Coordinates”, Second Edition, by George Paxinos and Keith B.J. Franklin. Clinical signs of epilepsy were observed for a few hours after the surgical procedure. Tissue samples were collected 24 and 72 h after model induction. Brains from uninjected mice and from mice injected in the same way but with PBS only were used as controls. The brain hemisphere with the site of injection is denoted as ipsilateral hemisphere (ipsi), whereas the opposite one is the contralateral hemisphere (contra).

### 2.3. Extraction of RNA and Proteins

To obtain RNA, animals were perfused with a saline solution. Tissues were weighed and then homogenized using an Ultra Turrax mechanical homogenizer (IKA, Staufen, Germany) in 1 mL of TRIzol LS Reagent (Gibco BRL, Invitrogen, Carlsbad, CA, USA). Homogenates were stored at −80 °C until use. RNA was isolated from the homogenates using the phenol–chloroform method described by Chomczynski and Sacchi [26]. After RNA isolation, its concentration was estimated using the photometric method (BioPhotometr Plus; Eppendorf Company, Vienna, Austria).

To obtain the proteins for the ELISA assay, the animals were perfused with a saline solution. Harvested organs were weighed and homogenized using an Ultra Turrax mechanical homogenizer (IKA, Staufen, Germany). Homogenization was performed in 1 mL of HEPES buffer, pH 7.4, containing HEPES—20 mM; EDTA—1.5 mM; benzamidine—0.5 mM; chicken egg ovoinhibitor—10 μg/mL; and PMSF (phenylmetylsulfonyl fluoride)—0.1 mM (Sigma-Aldrich, Poznan, Poland). Homogenates were then frozen and stored at −80 °C. Supernatants were obtained after centrifugation (20,000× *g*, 30 min, 4 °C; MPW; Warsaw, Poland).

### 2.4. Analysis of Gene Expression at the RNA Level by Real-Time PCR

A reverse-transcriptase reaction was performed with reverse-transcriptase enzyme M-MLV (Promega)—100 U/1 μg RNA. The reaction mix also contained RNaze-free water, buffer for reverse transcriptase (Promega, Madison, WI, USA), dNTPs (10 mM; Applied Biosystems, Waltham, MA, USA), 9-nucleotide random primers (nonamers; Sigma, Poznan, Poland) and RNaze inhibitor (Rnasin; Promega, Madison, WI, USA) 12.5 U/1 μg RNA. The reaction conditions were as follows: incubation of RNA and random nonamers for 3 min at 70 °C; 1 min incubation on ice and addition of remaining reagents; incubation for 1 h at 37 °C. After RT-PCR, samples with cDNA were stored at −20 °C until further experiments.

The analysis of gene expression was performed using a Corbett Real-Time PCR Machine Rotor Gene 3000 apparatus (Corbett Research, Sydney, Australia). The key enzyme used in this reaction was Taq polymerase with activity of 5 U/mL. The following were additional reaction components: buffer for polymerase, 25 mM MgCl_2_, 10 mM dNTPs, EvaGreen fluorescent dye (Biomibo, Warsaw, Poland), 10 μM primers specific to the amplified sequences and RNase/DNase-free water. For each reaction, 2 μL of cDNA derived from the reverse-transcription reaction was used. As a control histone, the H3 gene and reference RNA (QPCR Mouse Reference Total cellular RNA; Stratagene, La Jolla, CA, USA) were used. The reaction conditions were as follows: denaturation at 95 °C for 2 min; 45 cycles encompassing denaturation at 95 °C for 30 s; primer annealing for 30 s, elongation at 72 °C for 30 s.

### 2.5. Analysis of Gene Expression at the Protein Level by ELISA

The quantitative analysis of gene expression at the protein level was performed using the ELISA method with commercially available immunoenzymatic Quantikine Kits (R&D Systems, Minneapolis, MN, USA). Each set consisted of 96-well plates coated by the manufacturer, standard proteins used to prepare the standard curve, secondary and tertiary antibodies combined with the horseradish peroxidase enzyme, washing buffer and color substrate for peroxidase. The assay procedure was performed according to the protocol provided by the manufacturer. After stopping the color reaction, protein concentration was evaluated using a VICTOR2 Wallac 1420 photometric reader (PerkinElmer, Waltham, MA, USA) at a 450 nm wavelength, corrected at a 595 nm wavelength. All samples were analyzed in duplicates.

### 2.6. Quantitative Assessment of the Neurodegeneration Level Using the ELISA Method

The quantitative assessment of the intensity of neurodegeneration was performed using the ELISA method with primary antibodies directed against phosphorylated neurofilaments. The first step was the coating of a 96-well Maxisorb Microtitre plate (Nunc, Roskilde, Denmark) with monoclonal anti-NfH antibodies (SMI35R; Sternberger Monoclonals; Convance Princeton, NJ, USA) and followed by overnight incubation at 4 °C. Primary antibodies were diluted in carbonate buffer, pH 9.6. The next day, the samples and standard protein (neurofilament, 200 kD; Progen, Heidelberg, Germany) were added. As a secondary antibody, rabbit polyclonal anti-neurofilament 200 antibody (Sigma-Aldrich, Poznan, Poland) was used. As a tertiary antibody, swine antibodies against rabbit immunoglobulin conjugated with horseradish peroxidase (Dako, Glostrup, Denmark) were used. The final step was the addition of a color substrate for horseradish peroxidase, which was 3,3′,5,5′-tetramethylbenzidine (Sigma-Aldrich, Poznan, Poland). To stop the color reaction, 1 M HCl was used and the color photometric assessment was done using a VICTOR2 Wallac 1420 reader (PerkinElmer, Waltham, MA, USA). The analysis was performed at a 450 nm wavelength, corrected at a 595 nm wavelength. All samples were analyzed in duplicates and the concentration of NfH was determined by referring to the standard curve.

### 2.7. Detection of Neurodegeneration Localization Using Fluoro-Jade C Dye

The assessment of the localization and severity of neurodegeneration at the protein level was performed using Fluoro-Jade C fluorescent dye (Chemicon, Millipore, Warsaw, Poland). Animals were perfused with 4% buffered formalin solution and tissues samples were embedded in paraffin blocks. Sections of 10 μm in thickness were applied to polished Super Frost slides (Menzel-Glaser, Braunschweig, Germany). Before final staining, paraffin was removed by one-hour incubation at 60 °C and two ten-minute incubations in xylenes. Tissue was rehydrated in a series of alcohols. Fluoro-Jade C staining was performed according to the protocol provided by the manufacturer (Chemicon, Temecula, CA, USA). For nuclei staining, sections were counterstained using DAPI blue fluorescent dye (Sigma-Aldrich, Poznan, Poland). Then, the tissue was mounted and coverslipped using DPX (Sigma-Aldrich, Poznan, Poland).

Image Acquisition. For the analysis and acquisition of images, an AxioObserver A1 inverted microscope (Carl Zeiss Inc., Goettingen, Germany) was used. The following lenses made by Carl Zeiss Inc. were used: Plan-Achromat, 4×/0.10; A-Plan 10×/0.25 Ph1; LD A-Plan 20×/0.3; LD Plan-Neofluar 40×/0.6 Ph2 Korr. The images were obtained with a digital camera, AxioCamMRc5 (Carl Zeiss Group, Goettingen, Germany), attached to the microscope. For image acquisition, we used Axio-Vision Rel. 4.6 software (Carl Zeiss Group, Goettingen, Germany). After obtaining an image, no further processing was necessary.

Statistical Analyses. For the statistical analyses, nonparametric Mann–Whitney tests were used. For the correlation analyses, the Kendall tau test was used. A value of *p* < 0.05 was considered statistically significant.

## 3. Results

### 3.1. T-Cell-Marker Expression in the Brain in Animal Model of Epilepsy

**Figure 1:**
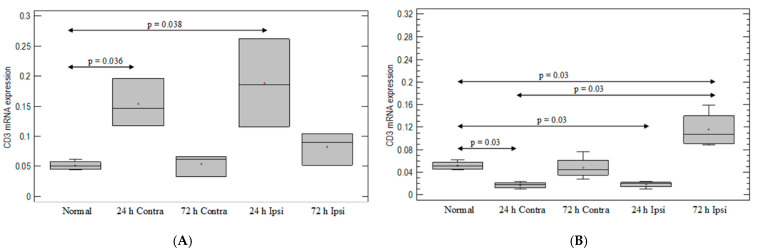
Expression of T-cell-line marker CD3 in mouse brain after (**A**) PBS injection and (**B**) KA injection. Brains were collected 24 h and 72 h postinjection. Ipsi—ipsilateral hemisphere; Contra—contralateral hemisphere; Normal—control animals without any injection.

A significant upregulation of the expression of the T-cell-line marker—CD3—was observed in PBS-injected brain (ipsilateral and contralateral hemispheres) on day 1, compared with untreated animals (*p* = 0.036 and *p* = 0.038, respectively; Mann–Whitney test) (Figure 1A). Interestingly, the injection of KA induced an almost three-fold decrease in CD3 expression on day 1 in both hemispheres as compared with the untreated group (*p* = 0.03; Mann–Whitney test) (Figure 1B). On day 3 after KA injection, increased CD3 marker expression was observed in the ipsilateral hemisphere, compared to untreated brain (*p* = 0.03; Mann–Whitney test), but not in the contralateral hemisphere (Figure 1B).

### 3.2. Monocyte-/Macrophage-Marker Expression in the Brain in Animal Model of Epilepsy

**Figure 2:**
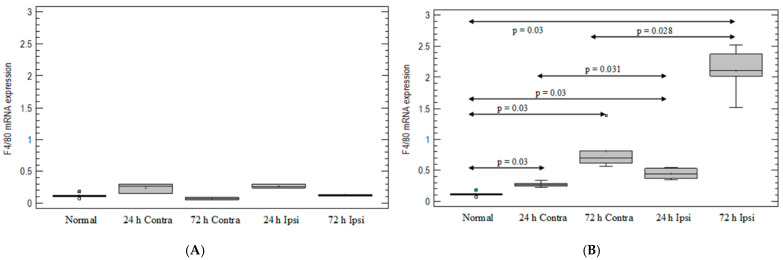
Expression of monocyte-/macrophage-cell-line marker F4/80 in mouse brain after (**A**) PBS injection and (**B**) KA injection. Brains were collected 24 h and 72 h postinjection. Ipsi—ipsilateral hemisphere; Contra—contralateral hemisphere; Normal—control animals without any injection.

After PBS injection, there was no statistically significant elevation in F4/80 at any point (Figure 2A). After KA injection, the expression of monocyte/macrophage lineage marker F4/80 was 4-fold and 20-fold elevated on day 1 and day 3, respectively, compared with untreated mouse brain (*p* = 0.03 for both; Mann–Whitney test) (Figure 2B). On day 1, an increased expression of F4/80 after KA injection was observed in the ipsilateral hemispheres compared with the contralateral hemispheres (*p* = 0.031; Mann–Whitney test) (Figure 2B) and compared with the PBS-injected group. On day 3 after KA injection, elevation was 3 times greater than in the contralateral hemispheres (*p* = 0.028; Mann–Whitney test) (Figure 2B) and 17-fold greater than in PBS-injected brain tissue (*p* = 0.03; Mann–Whitney) (Figure 2A,B).

### 3.3. Cytokine IL-1β Expression in the Brain in Experimental Model of Epilepsy

**Figure 3:**
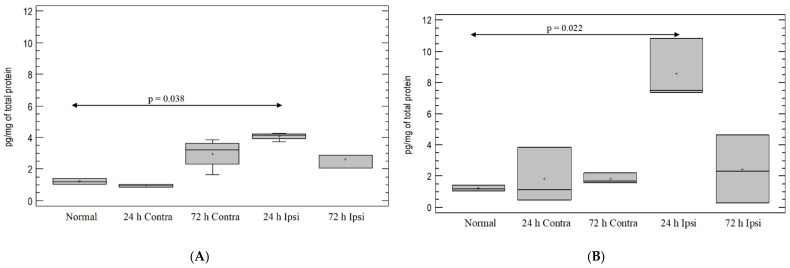
Cytokine IL-1β expression in mouse brain after (**A**) PBS injection and (**B**) KA injection. Brains were collected 24 h and 72 h postinjection. Ipsi—ipsilateral hemisphere; Contra—contralateral hemisphere; Normal—control animals without any injection.

A small upregulation of cytokine IL-1β was observed in the ipsilateral hemispheres one day from PBS injection as compared with the untreated group (*p* = 0.038; Mann–Whitney test) (Figure 3A). There was significant upregulation of IL-1β in the brain after KA injection on day 1. The increase was four-fold in ipsilateral hemispheres as compared with untreated control mice (*p* = 0.022; Mann–Whitney test) (Figure 3B). There was a statistically significant difference in IL-1β levels between the ipsilateral hemispheres after PBS and KA injections on day 1 (*p* = 0.036; Mann–Whitney test). On day 3, the amount of IL-1β protein decreased to a level comparable to that found in the untreated and PBS-treated groups (Figure 3A,B).

### 3.4. Expression of Chemokine CCL5 in Experimental Model of Epilepsy

**Figure 4:**
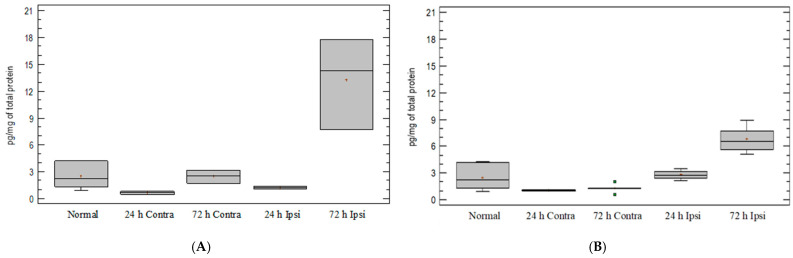
CCL5 expression in mouse brain after (**A**) PBS injection and (**B**) KA injection. Brains were collected 24 h and 72 h postinjection. Ipsi—ipsilateral hemisphere; Contra—contralateral hemisphere; Normal—control animals without any injection.

On day 1, there were no differences in CCL5 expression between the untreated group of mice and mice after PBS and KA injections (Figure 4A,B). The analysis of the CCL5 levels in KA-injected brain indicated that there were no significant differences between the ipsilateral hemispheres after KA and PBS injections. An upregulated expression of the CCL5 protein in the ipsilateral hemispheres was observed on day 3 after KA injection but comparable to that of the PBS-treated group (Figure 4A).

### 3.5. Expression of Chemokine CXCL2 in Experimental Model of Epilepsy

**Figure 5:**
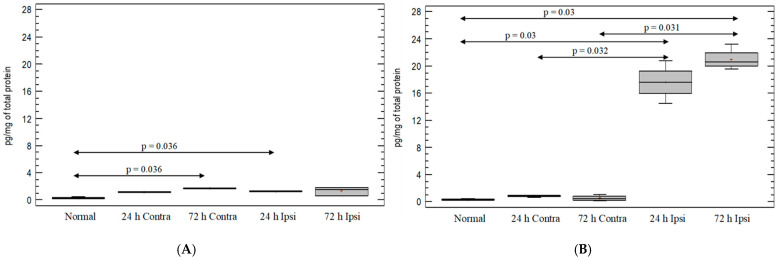
CXCL2 expression in mouse brain after (**A**) PBS injection and (**B**) KA injection. Brains were collected 24 h and 72 h postinjection. Ipsi—ipsilateral hemisphere; Contra—contralateral hemisphere; Normal—control animals without any injection.

A small upregulation of CXCL2 was observed in the PBS-injected control group as compared with untreated mice (Figure 5A). A massive upregulation of chemokine CXCL2 expression was observed in the ipsilateral hemispheres on day 1 and day 3 after injection of KA, compared with untreated mice (*p* = 0.03 for both time points; Mann–Whitney test) and the contralateral hemispheres (*p* = 0.032, *p* = 0.031; Mann–Whitney test) (Figure 5B). A statistically significant upregulation of CXCL2 expression in the ipsilateral hemispheres on day 1 and day 3 after KA injection was also observed in comparison with the PBS-treated group (*p* = 0.034, *p* = 0.033; Mann–Whitney test).

### 3.6. Expression of Chemokine CXCL12 in Experimental Model of Epilepsy

**Figure 6:**
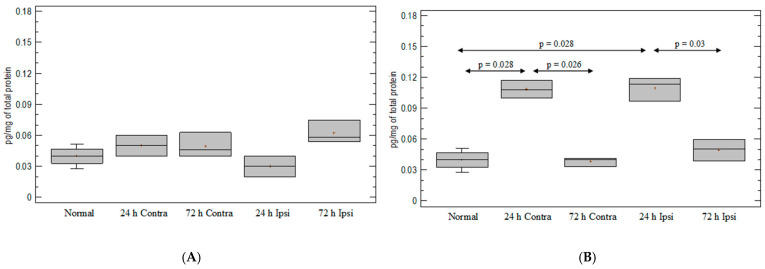
CXCL12 expression in mouse brain after (**A**) PBS injection and (**B**) KA injection. Brains were collected 24 h and 72 h postinjection. Ipsi—ipsilateral hemisphere; Contra—contralateral hemisphere; Normal—control animals without any injection.

After PBS injection, there was no statistically significant elevation on day 1 and 3 (Figure 6A). A large upregulation of CXCL12 chemokine expression was observed in both hemispheres on day 1 after KA injection in comparison with untreated mice (*p* = 0.028, *p* = 0.028; Mann–Whitney test) (Figure 6B). This upregulation was also greater than that observed in the hemispheres of the PBS-injected group (*p* = 0.036, *p* = 0.033; Mann–Whitney test). In the ipsilateral and contralateral hemispheres after KA injection, there was a decrease in CXCL12 expression on day 3 as compared with day 1 (ipsilateral hemispheres, *p* = 0.03; contralateral hemispheres, *p* = 0.026; Mann–Whitney test) (Figure 6B). On day 3, there were no differences in CXCL12 expression in KA-injected mice in comparison with untreated animals and the PBS-injected group.

### 3.7. Analysis of Intensity of Neurodegeneration in KA-Induced Experimental Epilepsy Model

**Figure 7:**
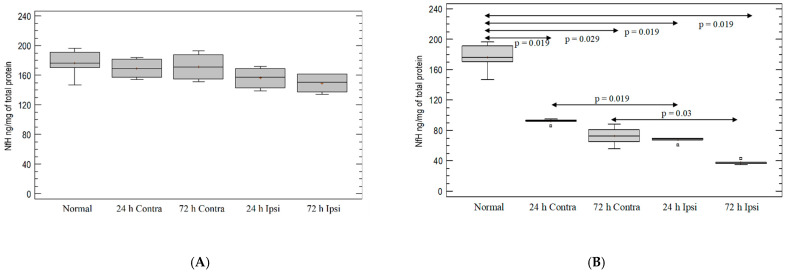
The level of phosphorylated neurofilaments in mouse brain after (**A**) PBS injection and (**B**) KA injection. Brains were collected 24 h and 72 h postinjection. Ipsi—ipsilateral hemisphere; Contra—contralateral hemisphere; Normal—control animals without any injection.

There was no decrease in NfH levels after PBS injection (Figure 7A). Statistically significant and ongoing neurodegeneration was observed in KA-injected hemispheres in comparison with untreated mouse hemispheres on day 1 (*p* = 0.019 for both hemispheres; Mann–Whitney test) and on day 3 (*p* = 0.029 for contralateral and *p* = 0.019 for ipsilateral hemispheres; Mann–Whitney test). The decrease in phosphorylated-neurofilament expression after KA injection was greater in the ipsilateral hemispheres than in the contralateral hemispheres on day 1 (*p* = 0.019) and day 3 (*p* = 0.03) (Mann–Whitney test; Figure 7B).

### 3.8. Analysis of Localization of Neurodegeneration in KA-Induced Experimental Epilepsy Model

**Figure 8:**
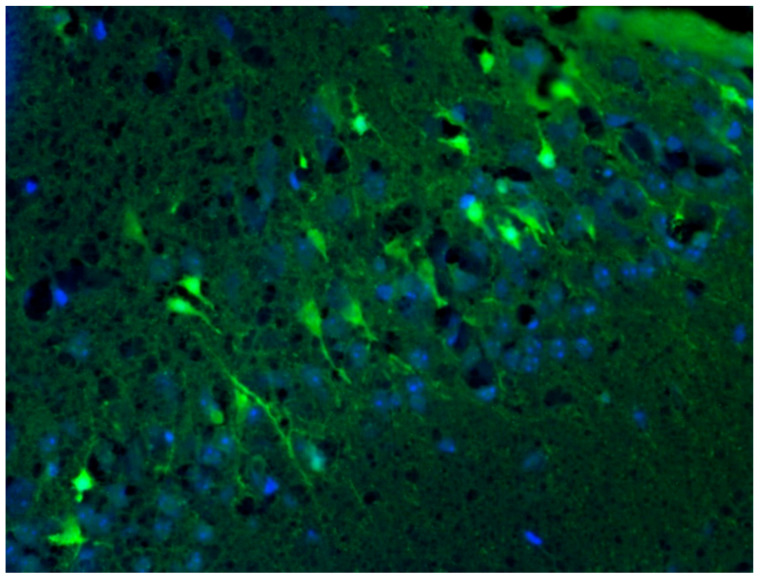
Localization of brain neurodegeneration in the site of KA delivery. Damaged neurons were stained using Fluoro-Jade C (light green color). Cell nuclei are counterstained with DAPI (blue color).

The localization of the neurodegeneration process was detected in KA-injected brain using Fluoro-Jade C staining. The injured neurons were abundant in tissue under exposure of KA (in Figure 8, representative injured neurons are marked by white arrows and cell nuclei are counterstained with DAPI in blue).

### 3.9. Correlation between Inflammatory Markers and Neurodegeneration in KA-Induced Epilepsy Model

**Figure 9:**
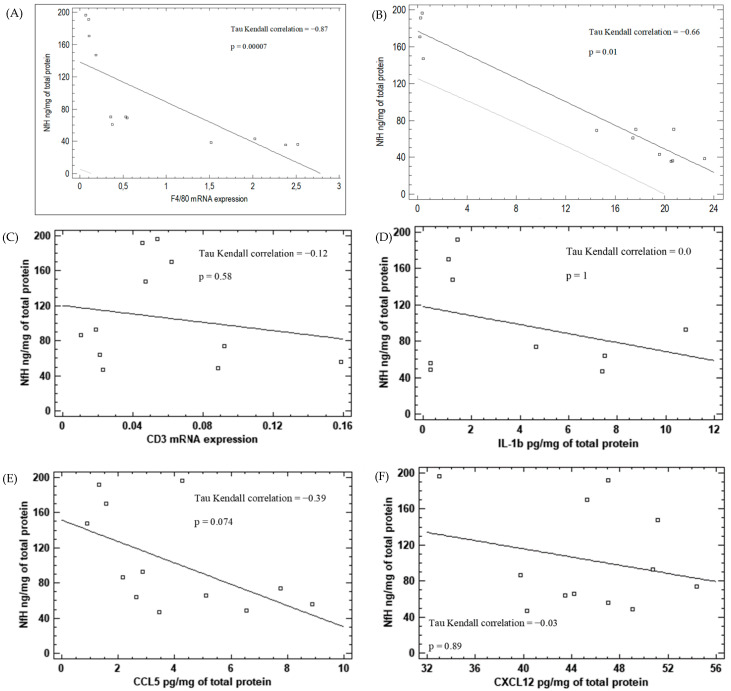
Correlation between monocyte/macrophage marker F4/80 (**A**), chemokine CXCL2 (**B**), T-cell-line marker CD3 (**C**), cytokine IL-1β (**D**), chemokine CCL5 (**E**), chemokine CXCL12 (**F**) and neurodegeneration marker NfH in KA-injected brain hemisphere.

We observed a negative correlation between the expression of monocyte/macrophage lineage marker F4/80 (Kendall tau = −0,87; *p* = 0.00007) as well as CXCL2 expression (Kendall tau = −0,66; *p* = 0.01) and the neurodegeneration process in the ipsilateral hemispheres after KA injection (Figure 9A,B, respectively). We did not observe a correlation between CD3, IL-1β, CCL5 and CXCL12 expression levels and neurodegeneration in neither the ipsilateral nor the contralateral hemispheres after KA injections (Figure 9C–F).

## 4. Discussion

Neuroinflammation is a CNS response to various insults, such as tissue damage, infection and immune reactions. This process encompasses the synthesis and release of numerous molecules with pro- or anti-inflammatory properties [27,28]. Neuroinflammation is typically associated with BBB dysfunction and peripheral immune-cell infiltration into the brain. In the epileptic brain, the presence of neutrophils and lymphocytes depends on the etiology of this disorder; however, macrophages are commonly observed [29,30]. Numerous studies indicated that the neuroinflammatory response in the brain is active during epileptogenesis and during acute symptomatic seizures, as well as in chronic epilepsy [27,31]. Moreover, the extent of this process differs among types of epilepsy with various etiologies, as well as among patients [32,33,34].

Signaling through small molecules is a hallmark for both the immune and nervous systems. Neurotransmitters affect their target cells through interaction with specific receptors. Cytokines are secreted by various cell types, such as endothelium, lymphocytes, astrocytes, microglia and neurons. Cytokine receptors in the CNS are present in neurons and glial-cell populations in discrete brain regions [35]. Cytokines are present at low concentrations in the CNS in a physiological state; however, their expression is highly elevated under pathological conditions. Although the pathophysiology of epilepsy is incompletely understood, the growing body of evidence suggests that there is a link between epilepsy and brain inflammation. Immune-mediated damage to the CNS is emerging as an important contributor to epileptogenesis, both through the inflammatory reactions and by induction of BBB damage [36]. Inflammatory reactions may enhance neuron excitability, increase neuronal death and alter the regeneration of neurons [10,37,38]. The magnitude of seizure activity impacts the inflammatory responses that follow seizures [39,40]. Indeed, it was observed in clinical and experimental data that neuroinflammation is a feature of hyperexcitable pathological brain tissue found in pharmacoresistant epilepsy [41].

Inflammatory processes in epilepsy may be studied by measuring the expression of the molecules, such as cytokines and chemokines, that mediate cell activation, differentiation and migration to the site of injury [42]. One of the widely studied cytokines in epileptic patients and animal epilepsy models is IL-1β. IL-1 cytokines are constitutively expressed at very low levels in the human CNS [6,43]. Under pathological conditions, their expression is increased in the brain. One of such conditions is the epileptic seizure, which enhances the expression of IL-1β, as well as IL-1Ra [44,45]. These molecules play important roles in excitotoxicity and oxidative stress [46,47,48]. IL-1β expressed by activated microglia and astrocytes enhances the release of glutamate from astrocytes and decreases its uptake, leading to neuronal hyperexcitability [49]. The deregulation of IL-1β expression alters the synaptic plasticity through the decreased LTP, which may result in neuronal dysfunction [50]. It was found, by Kothur et al. and Patterson et al., that IL-1β levels are rapidly elevated in glial cells within the first few hours of seizures [51,52].

Indeed, in our study, a significant increase in IL-1β protein levels was observed in mouse brain on day 1 after intracerebral KA injection as compared with PBS-injected animals and healthy controls. However, such upregulation was no longer present 3 days after model induction. These results may suggest the important role of this cytokine in the early phase of epileptogenesis. In studies conducted by Vezzani et al., increased concentrations of IL-1β were also observed 24 h after KA induction of status epilepticus (SE) in the rat hippocampus [53]. In various animal models of epilepsy, the pharmacological blockade of IL-1β through IL-1RA reduced seizures and signs of cellular injury, suggesting that the treatment of peripheral autoinflammation can be beneficial for intractable epilepsy [54,55,56]. Moreover, it was shown that the expression of IL-1R1, the receptor for IL-1β, was usually at very low levels in control brain tissue, but it was elevated in hippocampal neurons after seizures [57,58,59]. Strong immunoreactivity of IL-1β and its receptor was also observed in perivascular astrocytic endfeet and in the endothelial cells of the BBB during epileptogenesis [59]. The important role of IL-1β in epileptogenesis was proven in experiments with IL-1β administration in animal models of this disorder. It was observed that such administration worsened seizure activity and lowered the seizure threshold in rats and mice [44,60]. Moreover, the postnatal injection of IL-1β in a rat model of febrile seizures enhanced adult seizure susceptibility and hippocampal neuronal loss [61]. Studies utilizing pilocarpine-treated rats indicated that the early elevation of IL-1β expression preceded seizure onset, which suggests that the rise in IL-1β expression was not the result of seizure activity [54]. In KA-induced rodent epilepsy models, intrahippocampal injection of IL-1β worsened and prolonged both electrographic and behavioral seizure activity [44,53]. In SE induced by electric stimulation, the expression of proinflammatory cytokines, such as IL-1β, IL-6 and TNF-α, was elevated in the hippocampus within 1 day, similarly to what was observed in our experiments [45]. In animal models of epilepsy, the activation of IL-1β-related pathways was associated with neurodegeneration and BBB breakdown [58]. Contrary to these results, we did not observe any correlation between IL-1β levels and neurodegeneration assessed by NfH levels in our KA-induced epilepsy model.

The role for IL-1β in epileptogenesis was also proven in numerous clinical studies. In febrile seizures, increased levels of IL-1β, IL-6 and TNFα were detected in the CSF of patients [62]. Moreover, the increased expression of IL-1β in the CSF was also observed in pediatric epileptic patients, compared with the control group, which suggests the role of this cytokine in the initiation and progression of this disorder [63]. The increased expression of IL-1β was also observed in the brain tissue of TLE (temporal-lobe epilepsy) patients, compared with control tissue, especially in patients with hippocampal sclerosis [58,64,65]. The upregulation of IL-1β was also observed in patients with medial temporal-lobe epilepsy [66]. Contrary to these results, there were no significant differences in the IL-1β levels in the blood and CSF of patients with focal epilepsy [67]. Patients with focal drug-resistant epilepsy were shown to exhibit a pro-inflammatory disequilibrium in the IL-1β/IL-1RA ratio [68]. All of these results suggest that the deregulation of IL-1β levels may differ in various types of epilepsy, suggesting the contribution of different molecular pathways to their etiology.

Chemokines may modulate neuronal activity under physiological and pathological conditions by different mechanisms, e.g., modulation of voltage-dependent channels or increase in neurotransmitter release [69,70]. Thus, it is important to study the potential neuromodulatory role of chemokines in experimental and human epilepsy models. Recent evidence supports the role of chemokines in mediating immune-cell trafficking into the CNS during epilepsy [71]. Here, we assessed the expression levels of CCL5, CXCL2 and CXCL12 in a KA-induced model of epilepsy.

CCL5 controls neural plasticity and plays a role in inflammation and apoptosis via the interaction with CCR5 [72,73]. Mennicken et al. showed that there was an upregulation of CCR5 expression, one of the receptors for CCL5, after KA-induced seizures, specifically in hippocampal neurons and glial cells [74]. Kan et al. also observed such CCR5 upregulation in hippocampal astrocytes after pilocarpine-induced seizures [75]. RNA interference with CCR5 expression protected rats against KA-induced seizures by lowering hippocampal neuronal loss and macrophage/microglial infiltration [76]. Moreover, interference with CCR5 affected the seizure threshold. It was also shown that seizure activity induced the expression of chemokines CCL3, CCL4 and CCL5, which are the ligands for CCR5, facilitating BBB leakage. Thus, a decrease in CCR5 expression in circulating blood cells protected from BBB alterations and facilitated neurogenic repair [71].

Studies utilizing the KA-induced rat model of epilepsy showed the expression of CCR5 in the forebrain [74]. Moreover, Louboutin and coworkers observed that, in a KA-induced model, this receptor played an important role in seizure induction [71]. In this study, seizures induced a rise in CCL3 and CCL5 expression in brain vessels and increased BBB leakage and the migration of CCR5+ cells into the brain. These observations suggest that CCL3 and CCL5 expression in endothelial cells, together with the expression of CCR5 in leukocytes, is important for the control of vascular inflammation and leukocyte trafficking contributing to acute-seizure generation by excitotoxic agents [71]. It was confirmed, in a KA-induced seizure model, that CCL5 was implicated in the recruitment of inflammatory cells in the CNS [77]. Another study utilizing a KA-induced model of epilepsy confirmed that rats given KA showed an increased production of CCR5 ligands, which were observed in the microvasculature of the hippocampus [76,78]. Such increased production within blood vessels suggests that CCR5+ cells may be increased in the hippocampus of KA-treated animals. CCR5 was expressed mainly by lymphocytes, monocytes/macrophages, microglial cells and, to a lesser extent, neurons and astrocytes [71].

In our experimental model, we did not observe any significant differences in CCL5 protein levels between KA-injected animals and PBS-injected mice or healthy controls on day 1. However, the upregulation of this chemokine was evident on day 3 in KA-administered mice, compared with healthy animals. Our results agree with Louboutin et al.’s [71]. In their study, CCL5 and CCL3 were not detectable 6 h after KA injection; however, they were observed on day 3. On day 7, both chemokines were greatly increased after the injection of KA [71]. As CCR5 is expressed in various immune cells (as stated above), we assessed the expression levels of the lymphocyte marker (CD3) and monocyte/macrophage marker (F4/80) in the studied animal groups. CD3 levels were significantly decreased on day 1 after KA injection; however, its expression was upregulated on day 3 in mice with seizures as compared with healthy controls. F4/80 expression was significantly elevated on day 1 and day 3 in KA-injected mice, compared with the PBS-administered group and healthy animals. These results confirm the important role of monocytes/macrophages in epileptogenesis and suggest that lymphocyte recruitment is a secondary event during seizures in KA-induced epilepsy models. Moreover, we observed a correlation between neurodegeneration and the expression levels of F4/80, which further confirms the relevance of macrophages in the pathogenesis of epilepsy.

Clinical studies of epileptic patients also showed the deregulation of CCL5. CCL5 expression levels were elevated in TLE tissue, compared with autopsy control samples [79]. It was shown that CCL5 upregulation was observed only in TLE patients without sclerosis [79]. In Fiala et al.’s study, it was observed that CCL5 was expressed in lateral temporal cortical and hippocampal neurons in TLE patients but not in control cases [64]. Although this chemokine is usually found in cytoplasmic and extracellular locations, in TLE neurons, it was displayed in neuronal nuclei [64].

There is scarce information about the role of CXCL2 in patients and animal models of epilepsy. We observed that there was a significant upregulation of this chemokine both on day 1 and day 3 in KA-administered animals, compared with the PBS-injected group and healthy mice. Moreover, there was a correlation between the expression levels of CXCL2 and neurodegeneration. All of these results suggest that this chemokine plays an important role in epileptogenesis and seizure activity and needs further investigation to prove its role in this disease. CXCL12 is constitutively expressed in human CNS parenchyma in blood-vessel walls. Its increased expression observed in multiple-sclerosis lesions suggests that it plays a role in leukocyte transmigration into the CNS, which also implicates its role in epilepsy [80]. The increased expression of CXCR4 (CXCL12 receptor) was observed in the hippocampus of TLE patients mainly in microglia and a small population of astrocytes [37]. Such increased expression caused the intensified binding of CXCL12, thereby activating microglia to release TNFα, which potentiated glutamate release, resulting in neuronal hyperexcitability [37]. CXCL12 interaction with CXCR4 may play a proepileptic role. Two months after KA injection, one-week treatment with CXCR4 antagonist AMD3100 decreased the number and duration of spontaneous seizures [81]. It was shown that CXCL12 protein levels were raised after SE in the hippocampus [82]. After brain injury such as stroke or seizures, dead or damaged neurons and reactive glial cells, as well as endothelial cells, may express CXCL12. The increased expression of this chemokine was observed as early as 24 h after SE and could persist for 1 month [82]. The results obtained in our study partly confirm these observations. There was significant upregulation of CXCL12 on day 1 in KA-injected animals, compared with both control groups (PBS-injected and healthy mice). However, there were no differences in CXCL12 protein levels on day 3 among the studied experimental groups. Moreover, in the ipsilateral and contralateral hemispheres of animals subjected to KA administration, there was a downregulation of CXCL12 expression on day 3, compared with day 1.

The increasing body of clinical and experimental evidence suggests that peripheral inflammation can also influence epileptogenesis [83]. It was shown that leukocyte–endothelium interactions resulted in BBB alterations and subsequent infiltration of inflammatory cells into the CNS [84]. Indeed, epilepsy is typically accompanied by an increase in the number of leukocytes in the brain and this elevated cell transmigration is thought to lead to higher levels of neurodegeneration [84,85]. Thus, molecular markers of neuroinflammation may help to identify patients in whom aberrant inflammatory processes play a key role in epileptogenesis or in the maintenance of the epileptic state.

## 5. Conclusions

The major originality of our study is the finding of the increased expression of chemokine CXCL2 in the brain after KA injection. Moreover, we observed a significant correlation of this expression with neurodegeneration. To our knowledge, this finding is reported here for the first time. Its meaning is not known but we may speculate that CXCL2 could be one of the mediators of epileptogenesis in this model. Further studies are needed to confirm this hypothesis. In this project, we used the quantitative method of neurodegeneration measurement, which allowed us to perform reliable analyses of the correlations between the expression of inflammatory mediators and neuronal loss. This method is not used in experimental models of epilepsy. Currently, this method is introduced for the diagnosis of some neurological disorders, such as multiple sclerosis or dementia [86]. Most of the studies of KA-induced epilepsy models obtain results for cytokine expression and neurodegeneration based on staining methods [43,44,53]. This is in contrast to our study, as we obtained quantitative results based on the ELISA method. Moreover, only few reports analyze the correlation between cytokine levels and neurodegeneration.

## Data Availability

The data presented in this study are available on request from the corresponding author. The data are not publicly available due to internal regulations.
